# Use of a decision aid did not decrease decisional conflict in patients with carpal tunnel syndrome

**DOI:** 10.1186/s12891-017-1478-4

**Published:** 2017-03-21

**Authors:** Hyun Sik Gong, Jin Woo Park, Young Ho Shin, Kahyun Kim, Kwan Jae Cho, Goo Hyun Baek

**Affiliations:** Department of Orthopedic Surgery, Seoul National University College of Medicine, Seoul National University Bundang Hospital, 300 Gumi-dong, Bundang-gu, Seongnam-si, Gyeonggi-do 463-707 South Korea

**Keywords:** Carpal tunnel syndrome, Decision aid, Decisional conflict, Knowledge

## Abstract

**Background:**

Although a model for shared decision-making is important for patient-centered care, decisional conflict can emerge when patients participate in the decision-making. A decision aid is proposed to provide information and to involve patients more comfortably in the decision-making process. We aimed to determine whether a decision aid helps patients with carpal tunnel syndrome (CTS) experience less decisional conflict regarding their decision-making for surgery.

**Methods:**

Eighty patients with CTS were randomized into two groups. The test group was given a decision aid in addition to regular information and the control group regular information only. The decision aid consisted of a 6-min videoclip that explains diagnosis and information regarding surgery for CTS with other treatment options. We evaluated patients’ decisional conflict regarding surgery, knowledge about CTS, and symptom severity as measured by the Disabilities of Arm, Shoulder, and Hand (DASH) Questionnaire.

**Results:**

There was no difference in the decisional conflict scale (DCS) between both groups (*p* = 0.76). The test group had significantly better knowledge than the control group (*p* = 0.04). There was no correlation between the knowledge score and the DCS (*p* = 0.76). However, less severe symptoms were correlated with greater decisional conflict (*r* = −0.29, *p* = 0.02).

**Conclusions:**

We found that a decision aid does not reduce decisional conflict in patients with CTS, although it can help them be better informed. This study suggests that although a decision-aid is effective for patient education, doctor-patient communication should be more emphasized for patients with less severe symptoms, as they can have greater decisional conflict.

**Trial Registration:**

SNUBH Registry 1510/317-003 Registered November 13, 2015

**Electronic supplementary material:**

The online version of this article (doi:10.1186/s12891-017-1478-4) contains supplementary material, which is available to authorized users.

## Background

Carpal tunnel syndrome (CTS) is the most common peripheral neuropathy affecting the upper extremities, and it is characterized by a tingling sensation in the median nerve distribution and/or thenar muscle weakness. Although carpal tunnel release is a definitive treatment, there are several treatment options that depend on the severity of the symptoms and on the underlying cause [1, 2]. In addition, as the natural history of the condition remains unclear, questions may remain for both patients and physicians as to when and which treatment is most appropriate [3–5]. Therefore, the optimal decision-making for treatment depends not only on the probabilities of various outcomes with each strategy but also on the patient's relative preferences for the possible outcome states [6]. In this sense, support for a model of shared decision-making, in which clinicians and patients jointly make a decision, is a growing expectation for patients with CTS [7, 8]. However, decisional conflict can emerge from this decision-making process, when information about risks and benefits is not effectively communicated or discussed according to personal values [6, 7, 9].

Patients with CTS can receive information from a variety of sources, including the Internet, magazines, pamphlets, and TV programs. However, the quality and completeness of the information obtained from the above mentioned sources need to be improved [10, 11]. Therefore, a decision aid is proposed in order to provide information to patients and to involve patients in the surgical decision-making process [12]. Decision aids can take many forms, and the most common are comprised of a combination of written and oral information, personal counseling, videotapes, and interactive computer-based multimedia programs [6]. A study asking whether a directive to an Internet site enhances the doctor-patient interaction did not find any difference in patients’ knowledge and satisfaction levels in patients with CTS [13]. However, the effect that a decision aid has on patients’ decisional conflict in their decision-making has not been investigated. Therefore, we aimed to determine whether a decision aid helps patients with CTS experience less decisional conflict regarding their decision-making for surgery.

## Methods

### Participants

This study was approved by the ethical review board of our institution. We prospectively recruited eligible patients at an outpatient clinic in a tertiary referral setting who had been presented to a single hand surgeon for CTS diagnosis and treatment consultation. The inclusion criteria consisted of patients who had been referred to the hand clinic for the first time with symptoms and signs of CTS and had a positive electrodiagnostic test. We excluded those who had severe disease with thenar atrophy because we generally recommended surgery strongly in such cases, and doing so could have an effect on their level of decisional conflict. We also excluded those who had associated conditions, such as trigger digits, cubital tunnel syndrome, or cervical radiculopathy as well as those with worker’s compensation because these conditions could also affect their level of decisional conflict.

### Randomization

The surgeon recorded the history of the condition, performed a physical examination, and reviewed the electrodiagnostic test results. When the surgeon felt that patients were indicated for surgery, they were assessed for eligibility in the study and were then asked to participate. We obtained the written informed consent from all participants. The indication for surgery consisted of continuing or recurrent symptoms that did not improve after conservative treatment, such as splinting and/or corticosteroid injections. The surgeon explained the surgical procedure for open carpal tunnel release, the postoperative course, and the likely outcomes in a standard fashion. The patients who agreed to participate were assigned to one of two cohorts in a random order, as determined by using a random number generator (Windows Excel; Microsoft, Redmond, WA).

### Intervention

The patients in the intervention group were asked to view the decision aid while patients in the control group were not. The decision aid consisted of a 6-min videoclip using cartoons with up-to-date, evidence-based information regarding CTS diagnosis and information regarding surgery, as well as other available treatment options (Additional file 1: Movie S1). The aid met most of the quality criteria set by the International Patient Decision Aids Standards (IPDAS) Collaboration checklist [14]. Patients in both groups were finally asked whether they agreed to get surgery or whether they wanted other treatment options.

### Evaluation

When patients opted for surgery, a research assistant who had been blinded to the allocation evaluated the patients. The primary outcome was the decisional conflict with respect to surgery, which was measured by using the validated Decisional Conflict Scale (DCS) [9, 15]. This scale has 16 items and determines patients’ uncertainty in making a given health-related decision. Scores range from 0 (no decisional conflict) to 100 (severe decisional conflict). The scale contains one item to assess satisfaction, with the decision ranging from 0 (strongly agree) to 4 (strongly disagree). Statement format was administered.

The research assistant also measured patient knowledge about CTS by means of a paper test with 10 multiple-choice questions on the etiology, anatomy, purpose of surgical treatment, postoperative course, complications and other treatment options (Table 1). This test was developed by the authors and was not validated. The test score ranged from 0 to 10, with higher scores indicating better knowledge. Both the information given by the surgeon and the videoclip for the decision aid covered the content of the test. Both the DCS questionnaire and the knowledge questionnaire were administered once after the intervention with the decision aid.

**Table 1:** Carpal tunnel syndrome knowledge questionnaire (translated from Korean to English)

Question	Answer
1. What is the symptom of carpal tunnel syndrome? 1) tingling sensation 2) loss of sensation 3) muscle weakness 4) all	4
2. What is the main structure that is compressed at the carpal tunnel? 1) artery 2) nerve 3) ligament 4) all	2
3. What is the purpose of surgery? 1) to improve circulation 2) to relieve a nerve 3) to repair a ligament 4) all	2
4. What is the usual method of anesthesia for the surgery? 1) no anesthesia 2) local anesthesia 3) general anesthesia	2
5. What is cut during the surgery? 1) artery 2) nerve 3) ligament 4) all	3
6. When do I have the sutures removed? 1) 5 days 2) 2 weeks 3) 1 month	2
7. When do I usually regain the grip strength? 1) 5 days 2) 2 weeks 3) 1 month 4) 3–6 months	4
8. How long can I have pain around the scar? 1) 5 days 2) 2 weeks 3) 1 month 4) 3–6 months	4
9. What is a common result of not treating carpal tunnel syndrome for long? 1) no circulation at finger tips 2) thumb muscle weakness 3) limitation of finger joint motion 4) all	2
10. What is an alternative, although not a definitive, treatment to surgery? 1) splinting 2) local anesthetics injection 3) shock wave therapy 4) electrical stimulation	1

The research assistant also recorded patients’ characteristics in terms of education level, comorbidities (such as diabetes mellitus, thyroid disease or chronic renal disease), history of a previous operation, symptom duration, and symptom severity as measured by the Disabilities of the Arm, Shoulder, and Hand (DASH) questionnaires [16].

### Statistical analysis

The sample size was determined by using the DCS as the primary outcome. A previous study regarding DCS in patients with an asymptomatic abdominal aneurysm exhibited a mean DCS ranging from 15 to 35 and a standard deviation ranging from 13 to 22 [17]. We assumed a standard deviation of 15 points and aimed to detect a significant difference of 12 points between the groups on the 100-point scale of the DCS (effect size of 0.8). The power analysis indicated that a Student’s *t*-test with a sample size of 31 patients per group would provide 90% statistical power to detect an effect of this size between groups at a significance level of 0.05.

To compare the two groups, a Pearson chi-square or Fisher exact test was used for categorical variables while Student’s *t*-test was used for continuous variables. We also performed correlation analyses to identify relationships between variables in the overall patient population. All statistical analyses were performed using SPSS (version 19.0; SPSS, Chicago, Illinois), and p values < 0.05 were considered to be significant.

## Results

**Fig. 1:**
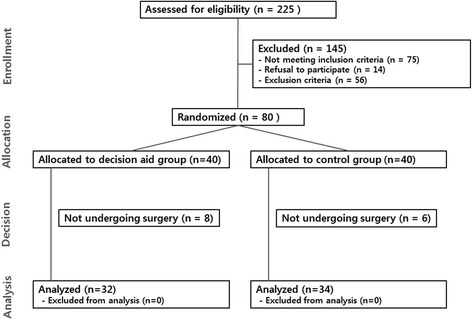
A CONSORT flow diagram for enrollment, allocation, and analysis

Two hundred and twenty five patients were assessed for eligibility and 80 patients were randomized to either a decision aid group (*n* = 40) or a control group (*n* = 40). Thirty-two patients from the decision aid group and 34 from the control group opted for surgery and were included in the final analysis (Fig 1). Both groups were comparable in terms of demographic characteristics, education, comorbidities, history of a previous operation, symptom duration, and symptom severity assessed with DASH scores (Table 2).

**Table 2:** Baseline characteristics and outcome measures in both groups

	Decisional aid group	Control group	*p* value
	(*n* = 32)	(*n* = 34)	
Age in years (SD)	53 (10)	52 (9)	0.69
Male (%)	6 (19%)	8 (24%)	0.45
Symptom duration in months (SD)	34 (25)	32 (28)	0.74
Bilateral involvement (%)	12 (38)	11 (32)	0.59
Less than a high school education (%)	14 (43)	16 (47)	0.79
Comorbidities (%)	13 (41)	13 (38)	0.84
History of a previous operation (%)	7 (22)	9 (26)	0.66
DASH scores (SD)	41 (13)	40 (15)	0.73
Knowledge about CTS (SD)	7.4 (1.5)	6.6 (1.5)	**0.04**
Decisional conflict scale (SD)	22 (15)	23 (16)	0.76
Satisfaction with the decision (SD)	1.8 (0.8)	1.7 (0.6)	0.80

*SD* standard deviation; Boldface means a significant value

The decisional conflict scores did not differ between the decision aid and the control groups (22 vs. 23, *p* = 0.76). The scores of the patients’ satisfaction with their decisions also did not differ between the two groups (1.8 vs. 1.7, *p* = 0.80). However, the decision aid group patients had greater knowledge of CTS than those in the control group (7.4 vs. 6.6; *p* = 0.04). Both the DCS and knowledge scores demonstrated a normal distribution.

An analysis of all patients in both groups revealed that there was no correlation between decisional conflict and knowledge scores (*r* = 0.03, *p* = 0.76). However, a negative correlation was found between decisional conflict and DASH scores (*r* = −0.29, *p* = 0.02), meaning that those with less severe symptoms had greater decisional conflict. No significant correlation was found between decisional conflict and the other variables, such as age, education, comorbidities, history of a previous operation, and symptom duration.

## Discussion

Patients with CTS can be informed through a variety of sources and several treatment options are available. However, the effect that a decision aid has on patients’ decisional conflict regarding surgery on their knowledge of their condition regarding surgery is not known. This study demonstrates that although patients with CTS who received a decision aid had greater knowledge of their condition, the decision aid did not significantly reduce decisional conflict or increase satisfaction with the decision when compared to patients who were given regular information by the surgeon. However, this study does show that patients with less severe symptoms had greater decisional conflict regarding surgery.

In the present study, the decision aid did not reduce decisional conflict, which is contrary to what was reported in previous reviews [12, 18]. However, a few studies likewise reported no difference in decisional conflict, such as in studies of patients with an asymptomatic abdominal aortic aneurysm [17], or patients with advanced colorectal cancer who were considering chemotherapy [19]. The reasons for which no differences were found were not presented in these studies [17, 19]. O’Connor et al. reported that decisional conflict scores of 25 or lower are associated with a follow-through with decisions, whereas those exceeding 38 are associated with a delay in decision making [20]. Therefore, the low decisional conflict scores in both groups obtained by the current study could indicate that patients with CTS may already feel comfortable with the usual care, resulting in a ceiling effect for an added decision aid. This could also be the reason for which no difference in satisfaction was reported for the decision making process [18]. The analysis of all patients revealed, however, that patients with more severe symptoms had less decisional conflict. This is consistent with the previous finding that the severity of subjective symptoms is the most important reason of all factors that affect the willingness to undergo carpal tunnel release [21].

In this study, the patients in the decision aid group had greater knowledge than those in the control group, which is consistent with observations from previous studies on decision aids for patients facing a surgical treatment decision for other conditions [12]. Furthermore, a recent Cochrane review on decision aids for people facing health treatment or screening decisions revealed that 42 out of 56 studies comparing decision aids to usual care indicated that people exposed to decision aids had higher average knowledge scores, with a mean of 13.34% (95% Confidence interval 11.17 to 15.51) [18]. The review also revealed that people exposed to detailed decision aids had higher knowledge scores, but the effect was small with a mean difference of 5.52%. On the contrary, Aung et al. found that a directive to an Internet site did not enhance patients’ knowledge of CTS [13]. They tested the patients’ knowledge 3–4 weeks after the first visit when they recommended the web site, which could make the patients forget the information. In addition, the information on the web site could be difficult to read compared to the cartoon-based video format used in the current study. The expected knowledge results are contrary to the survey by Hageman et al., which found that patients with CTS favored web-based information more than surgeons, who valued a video format for information [22]. Future studies may determine which type of decision aid can better increase knowledge, and whether the knowledge is maintained and helps patients cope with postoperative recovery.

The decision aid used in this study was a series of cartoons. There are a few studies supporting the use of cartoon animations to inform patients. Tou et al. showed that preoperative 2D animation information is an effective medium for delivering information to patients undergoing bowel surgery and can reduce anxiety related to surgery [23]. Imamura et al. also showed that an internet-based cognitive behavioral therapy program in Manga format was effective in improving depression in the general working population [24]. We consider that cartoons can show the important points about anatomy and surgery more simply than real operative photographs. However, the optimal medium for production of decisional aid remains unknown.

There are several limitations to this study. First, this study was performed in a tertiary referral setting and all of the patients had been referred by other physicians. Therefore, the doctor patient-relationship with their previous physician or the information that they were previously provided could have affected the patients in our study. Second, both the patients and the physician were not blinded to the allocation. The patients knew the aims of the study, and those allocated to the decision aid group could have participated more actively in the knowledge test, a phenomenon known as the Hawthorne effect [25]. Third, the knowledge questionnaire was not a validated form, as there is currently no validated survey to test patients’ knowledge about CTS. However, the scores showed a normal distribution, and the chances of getting answers correct by guessing would be low since the test was composed of multiple-choice questions.

## Conclusions

This study found that a decision aid does not reduce decisional conflict in patients with CTS, although it can help the patients be better informed. This study suggests that although a decision-aid is effective for patient education, doctor-patient communication should be more emphasized for patients with less severe symptoms, as they can have greater decisional conflict.

## Additional file

Additional file 1: Movie S1. Description of data: The file is a 6-min videoclip using cartoons regarding carpal tunnel syndrome diagnosis and information regarding surgery, as well as other available treatment options. (WMV 26993 kb)

## Abbreviations

CTS: Carpal tunnel syndrome
DASH: Disabilities of arm, shoulder, and hand
DCS: Decisional conflict scale
IPDAS: International patient decision aids standards
